# Learning naturalistic driving environment with statistical realism

**DOI:** 10.1038/s41467-023-37677-5

**Published:** 2023-04-11

**Authors:** Xintao Yan, Zhengxia Zou, Shuo Feng, Haojie Zhu, Haowei Sun, Henry X. Liu

**Affiliations:** 1 Department of Civil and Environmental Engineering, University of Michigan, Ann Arbor, MI USA; 2 University of Michigan Transportation Research Institute, Ann Arbor, MI USA; 3 Mcity, University of Michigan, Ann Arbor, MI USA; 4 Present Address: School of Astronautics, Beihang University, Beijing, China; 5 Present Address: Department of Automation, Tsinghua University, Beijing, China

**Keywords:** Civil engineering, Mechanical engineering

## Abstract

For simulation to be an effective tool for the development and testing of autonomous vehicles, the simulator must be able to produce realistic safety-critical scenarios with distribution-level accuracy. However, due to the high dimensionality of real-world driving environments and the rarity of long-tail safety-critical events, how to achieve statistical realism in simulation is a long-standing problem. In this paper, we develop NeuralNDE, a deep learning-based framework to learn multi-agent interaction behavior from vehicle trajectory data, and propose a conflict critic model and a safety mapping network to refine the generation process of safety-critical events, following real-world occurring frequencies and patterns. The results show that NeuralNDE can achieve both accurate safety-critical driving statistics (e.g., crash rate/type/severity and near-miss statistics, etc.) and normal driving statistics (e.g., vehicle speed/distance/yielding behavior distributions, etc.), as demonstrated in the simulation of urban driving environments. To the best of our knowledge, this is the first time that a simulation model can reproduce the real-world driving environment with statistical realism, particularly for safety-critical situations.

## Abstract (web summary)

Simulation of naturalistic driving environment for autonomous vehicle development is challenging due to its complexity and high dimensionality. The authors develop a deep learning-based framework to model driving behavior including safety-critical events for improved training of autonomous vehicles.

## Introduction

Autonomous driving technologies are revolutionizing the future of transportation systems in unprecedented ways and speeds. However, safety remains the key challenge for the development and deployment of highly automated driving systems^1,2^. Simulation provides a controllable, efficient, and low-cost venue for both developing and testing autonomous vehicles (AV)^3,4^. But for simulation to be an effective tool, statistical realism of the simulated driving environment is a must^2,4–7^. In particular, the simulated environment needs to reproduce safety-critical encounters that AV might face in the real world with distribution-level accuracy. Unfortunately, the real-world naturalistic driving environment (NDE) is spatiotemporally complex and highly interactive. Therefore, how to achieve statistical realism for such simulators is a long-standing problem in the field.

In recent years, great efforts have been made in developing simulators for autonomous driving systems. Thanks to rapid advances in artificial intelligence (AI), computer vision and graphics, and high-performance computing devices, accurate vehicle dynamics, photorealistic rendering, and realistic sensor simulation are now being realized and accessible. Some well-known simulators include Intel’s CARLA^8^, Google/Waymo’s CarCraft^9^ and SimulationCity^5^, Tesla’s simulator, Microsoft’s AirSim^10^, NVIDIA’s DRIVE Sim^11^, Baidu’s AADS^3^, and Cruise’s simulator, etc. Despite the above efforts and advancements, these simulators mainly focus on the fidelity of the vehicle rather than the driving environment, especially for the background road user behavior. The behaviors of background agents are either replayed from logged data or simulated using oversimplified heuristic rules, which leaves a significant gap between the simulation and the real-world driving environment.

The key to high-fidelity NDE is accurate modeling of human driving behavior. Microscopic traffic simulators, which mimic the interactive agent behaviors through a combination of physics-driven models and hand-crafted rules, such as car-following models^12,13^, lane-changing models^14,15^, gap-acceptance models^16^, etc., have been studied and developed in the transportation engineering domain for decades. Some well-known traffic simulators are SUMO^17^, VISSIM^18^, and AIMSUN^19^. Due to the limited capability of the underlying parametric models and manually encoded rules, the model fidelity is constrained. Many attempts have been made by using neural networks^20–25^, Markovian-based models^6,26^, Bayesian networks^27,28^, and game theory^29^, etc., to achieve better performance in modeling specific behaviors (e.g., car-following) or specific scenarios (e.g., unprotected left turn). However, they can hardly be generalized and scaled to model complex urban environments and highly interactive scenarios.

The focus of this study is to build a high-fidelity simulator that is statistically representative of real-world driving environments, particularly for those long-tail safety-critical events. Especially, we aim to produce safety-critical events with distribution-level accuracy, including both crashes and near-misses, which are critical for training and testing AVs. This differentiates our proposed NeuralNDE model from most existing simulators based on imitation learning (including generative adversarial imitation learning)^30–36^, where statistical realism is hardly considered and cannot be achieved. For example, the crash rates of these simulation environments are significantly higher (e.g., SimNet^33^) than that of real-world traffic. Moreover, these methods can only generate short-time simulations in the order of a few seconds (e.g., D2Sim^36^), which limits the capability of full-length trip training and evaluation of AVs. To reproduce high-fidelity safety-critical events, there are also methods proposed based on real-world event reconstruction. For example, the researchers constructed the simulation environment based on real-world fatal collision events from various data sources including police reports^37^. However, it may be difficult to reconstruct near-miss events using this method since the information needed for reconstruction is usually not available. Therefore, these reconstruction-based methods and our learning-based method serve to complement each other when building high-fidelity simulators.

**Fig. 1:**
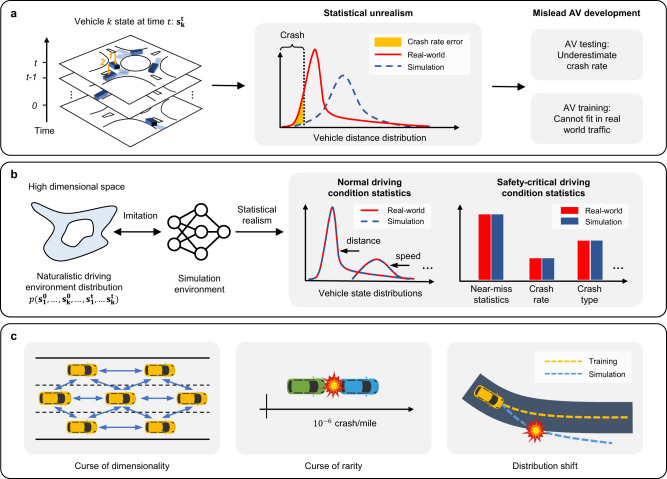
Modeling naturalistic driving environment with statistical realism. **a** Statistical errors in simulation may mislead AV development. **b** The underlying naturalistic driving environment distribution is highly complex and in a high-dimensional space since it involves multiple agents and long-time horizons. The simulation environment needs to achieve statistical realism, i.e., distribution-level accurate statistics regarding human driving behaviors in both normal and safety-critical driving conditions. **c** Major challenges for modeling multi-agent interaction behaviors and constructing naturalistic driving environments. The challenges include the “curse of dimensionality” for multi-agent highly interactive behaviors, the “curse of rarity”^38^ of safety-critical events in the real world, and the “distribution shift” for long-time simulations.

The lack of statistical realism for simulation can potentially mislead AV development in both training and testing. An illustration example is shown in Fig. 1a. Consider a roundabout environment that includes multiple vehicles. At time $t$, a vehicle (vehicle 1) is circulating, and another vehicle (vehicle 2) is about to enter the roundabout. Their potential future positions are denoted by shaded blue areas, and they have a probability to collide if vehicle 2 fails to yield. Assume the distance between the two vehicles in the real world follows certain distribution as shown by the red curve and the simulated results are the dashed blue curve. This statistical difference, i.e., distribution inconsistency between the real world and simulation, will lead to an underestimation of vehicle crash rate and therefore provide optimistic estimates of AV safety performance. Also, since the distance between vehicles in the simulation environment is not consistent with the real world, an AV agent trained in it might not fit in real traffic due to the large sim-to-real gap. In real-world driving environments, instead of two agents, multiple human drivers are continuously interacting with each other and their states are progressively evolving for a long-time horizon. Therefore, the underlying joint NDE distribution is extremely complex and in a very high-dimensional space as shown in Fig. 1b. The goal of NDE modeling is to achieve distribution-level accuracy under both normal driving and safety-critical situations. Therefore, a wide range of environment statistics, for example, vehicle speed and distance distributions, crash rate, crash type and severity distributions, near-miss measurements, etc., need to be consistent with the real world.

The challenges of modeling NDE with statistical realism mainly come from three aspects as shown in Fig. 1c. The first challenge is from the “curse of dimensionality”. The real-world driving environment is highly interactive and spatiotemporally complex with large numbers of road users and long-time horizons, which make NDE modeling a very high-dimensional problem. The second challenge is from the “curse of rarity”^38^. Since safety-critical events (e.g., crashes) rarely happen in the real-world driving environment (on average ${10}^{-6}$ crashes per driving mile for human drivers^39^), modeling such rare events in high-fidelity requires an extremely high precision of the microscopic behavior. The compounding effects of the “curse of rarity” on top of the “curse of dimensionality” in the real world NDE will make it even more challenging^38^. The third challenge is from the “distribution shift”^30^, which is particularly critical for learning-based simulators. Short-term and small modeling errors may accumulate both in space and time, which might lead to out-of-distribution behaviors like frequent offroad, unrealistic collision, or even the collapse of the entire simulation. Moreover, due to the highly interactive nature of the driving environment, unrealistic behaviors of a single agent will impact and propagate to all agents in the simulation.

**Fig. 2:**
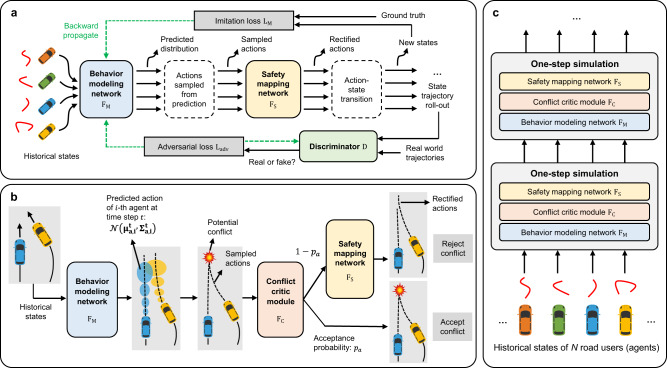
The proposed NeuralNDE framework. **a** The framework and training pipeline of the NeuralNDE. Two types of loss, i.e., imitation loss and adversarial loss, will be backpropagated to train the behavior modeling network to learn multi-agent interactive behaviors. The adversarial loss will also be used to train the discriminator for distinguishing between real-world and simulated trajectories. The safety mapping network is pretrained and fixed when training the behavior modeling network and the discriminator jointly. **b** Demonstration of the behavior modeling network, conflict critic module, and safety mapping network during inference time. The behavior modeling network predicts the action distribution of each vehicle. In safety-critical situations, predictions can be inaccurate and lead to unrealistically high crash probability, because the training data is overwhelmingly from normal driving situations. Therefore, the safety mapping network will guide vehicle behavior and rectify their actions in such situations. With a certain probability, we will accept the generated conflict without passing it to the safety mapping network. The acceptance probability is trajectory-dependent and will be calibrated to fit ground-truth safety-critical statistics (e.g., crash rate and crash type distribution). Therefore, the conflict critic module controls the occurring frequencies and patterns of dangerous driving behavior during the simulation. **c** Illustration of the simulation process. The historical states of all road users will pass through the behavior modeling network, conflict critic module, and safety mapping network to generate the states at the next timestep. This process will proceed progressively to simulate the driving environment.

In this paper, we solve this long-standing problem by developing NeuralNDE—a novel deep learning-based framework for simulating Naturalistic Driving Environment with statistical realism. The overview of the proposed framework is shown in Fig. 2a. We frame the simulation modeling under an imitation learning paradigm with deep neural networks under the supervision of large-scale real-world demonstration. The behavior modeling network takes in all road users’ past states within a historical time window as input and predicts their joint distribution of future actions. We leverage the recent advances in fundamental models (e.g., GTP^40^ and BERT^41^) and use Transformer as the backbone of the behavior modeling network to characterize multi-agent interaction behaviors. The behavior modeling network can achieve distribution-level accuracy in normal driving conditions, however, it cannot achieve such accuracy in safety-critical conditions, due to the rarity of safety-critical events in the training data, which will lead to inaccurate statistics like unrealistically high crash rates. To tackle this issue, a conflict critic mechanism is introduced during the inference time as shown in Fig. 2b. It will monitor the generated trajectories, and if there is a potential conflict, there is a certain probability to accept vehicles performing dangerous behavior, which makes NeuralNDE capable of realizing accurate safety-critical statistics. Otherwise, the generated behaviors will be guided and rectified by the safety mapping network to resolve the conflict. The differentiable safety mapping network is a neural mapper pretrained from physics and driving rules to map unsafe behaviors to a feasible domain of safety. To further overcome the distribution shift issue, we integrate the generative adversarial training as in GAN^42^ and GAIL^43^, where a discriminator is introduced to be jointly trained with the behavior modeling network. During the simulation process, as shown in Fig. 2c, the state of all road users will be updated based on the behavior modeling network, conflict critic module, and the safety mapping network in each simulation step to autoregressively generate the simulation environment.

To demonstrate the effectiveness of our approach, we construct two multi-lane roundabout environments located in the US and Germany, respectively, using real-world data. An illustration video is provided in Supplementary Movie 1. The simulated environment is validated to be statistically accurate with the real world, including vehicle instantaneous speed, distance, and yielding behavior. More importantly, the proposed NeuralNDE can achieve accurate safety-critical statistics including both crash and near-miss measurements, for example, crash rate, crash type, crash severity, post-encroachment time (PET^44^), etc. The fidelity of NeuralNDE-generated crash events is further validated against real-world crash videos and police crash reports. To the best of our knowledge, this is the first time that a simulation environment can systematically reproduce the real-world driving environment with statistical realism, particularly for those long-tail safety-critical events that are critical to AV safety. In addition, the proposed environment can perform long-time (hour-level) simulation, where the AV under training or testing can continuously interact with background vehicles. The proposed NeuralNDE should be readily integrated with different high-fidelity AV simulators, for example, CARLA^5^, which focuses on photorealistic rendering and sensor simulations, to provide a realistic traffic environment. Furthermore, it should be noted that the proposed NeuralNDE model can be used for other safety-related applications other than AV training and testing. For example, the proposed NeuralNDE model can be used to estimate the safety performance of a traffic facility under different traffic flow conditions.

## Results

### Dataset

**Fig. 3:**
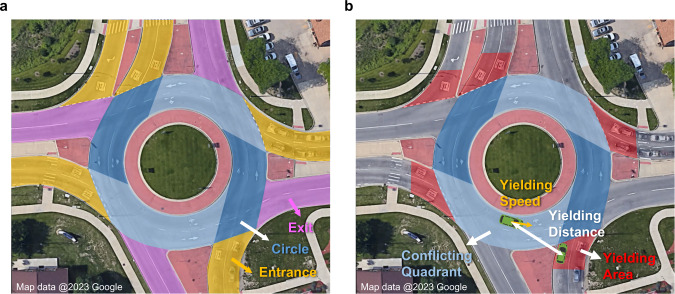
Illustration figure of the studied location. **a** Illustration figure of the Ann Arbor roundabout. **b** Illustration figure of the yielding area. The vehicle in the red yielding area is the yielding vehicle. The vehicle in the corresponding conflicting quadrant is the conflicting vehicle. The yielding distance and yielding speed are demonstrated in the figure.

Roundabout is an important and challenging urban driving environment for AVs. We validate our model using a real-world dataset collected from a two-lane roundabout located at State St. and W Ellsworth Rd. intersection, Ann Arbor, Michigan, USA (abbreviated as AA dataset). The illustration figure of this two-lane roundabout is shown in Fig. 3a. This is a busy roundabout with a large traffic volume and the fourth-highest crash rate in Michigan^45^. A roadside perception system^46,47^ is deployed for real-time traffic object detection, localization, and tracking to collect all vehicle trajectory information (e.g., position, heading) within the roundabout at 2.5 Hz. The AA dataset includes both detailed normal and safety-critical driving conditions data. The safety-critical events data, which includes crash event trajectories, crash videos, police crash reports, etc., are crucial for providing safety-critical statistics ground-truth to validate the simulation fidelity. To the best of our knowledge, real-world safety-critical rare-event data are not available in most existing public datasets, however, they are essential for constructing and validating the performance of generated simulation environments. More details about the dataset can be found in Supplementary Section 1a.

We also use an open dataset, rounD^48^, which is collected at a two-lane roundabout located at Neuweiler, Aachen, Germany, to further demonstrate NeuralNDE performance in normal driving conditions. Due to space limits, we will use results on AA datasets to illustrate the performance, and the results using the rounD dataset can be found in Supplementary Section 2b.

### Experiment settings

The proposed NeuralNDE simulator is first initialized with a randomly sampled trajectory clip of 2 seconds with all agents following their logged trajectories. Then, all agents’ behaviors are controlled by NeuralNDE. At each simulation time step, new vehicles will be generated in each entry lane by following a Poisson process whose arrival rate is calibrated using the dataset. Also, vehicles will leave the road network when reaching exit areas. Each simulation episode lasts for 3600 seconds with a simulation resolution of 0.4 seconds. If a crash happens, the simulation will be terminated early. We use ~15,000 simulation hours of data to validate the statistical realism of the NeuralNDE, where all data are used for calculating crash-related metrics and 100 hours of data are used for other metrics. We conducted the experiments on the University of Michigan’s Great Lakes High-Performance Computing (HPC) cluster using 1000 cores and 2000 GB RAM. It took around 1440 seconds of real-world time to conduct 3600 seconds of simulation. Therefore, the simulation speed ratio (simulation time/real-world time) is ~0.4. More details about experiment settings can be found in Supplementary Section 1b.

### Evaluation metrics

To evaluate the fidelity and statistical realism of the proposed NeuralNDE, a suite of statistical metrics is examined, with both normal and safety-critical driving behaviors. The metrics include (1) vehicle instantaneous speed distribution; (2) vehicle distance distribution; (3) vehicle yielding distance and yielding speed distributions; (4) vehicle crash rate; (5) vehicle crash type distribution; (6) vehicle crash severity distribution; (7) vehicle post-encroachment time (PET) distribution. Detailed definitions for the metrics are introduced in the following paragraphs.

The instantaneous speed distribution is collected when vehicles travel in the roundabout circle. The speed is calculated using the Euclidean distance traveled between two timesteps divided by the simulation time resolution. To measure the distance between two vehicles, each vehicle is approximated using three circles with an equal radius as shown in Supplementary Fig. 1a. Vehicle distance is defined by the nearest circle centers of two vehicles. We use $r=1.0$ meters and $l=2.7$ meters in this study.

A vehicle is considered to yield if it reaches a running stop, i.e., speed smaller than 5mph, in the yielding area of each entry as shown in Fig. 3b. Vehicles in the corresponding circle quadrant as shown in Fig. 3b are conflicting vehicles for the vehicle in the yielding area. The vehicle yielding distance is the Euclidean distance between (1) the yielding vehicle at the entrance and (2) the nearest conflicting vehicle in the roundabout. The speed of the closest conflicting vehicle is recorded for the vehicle yielding speed distribution.

Two agents are considered in a crash if their rectangle bounding boxes overlap. The crash rate is calculated by the number of collisions divided by the total travel distances of all vehicles. The crash type is adopted from the definition of the National Highway Traffic Safety Administration^49^. More information can be found in Supplementary Section 1c.

We use the change in velocity (Delta-V), a widely used metric to estimate occupant injury risk to measure the simulated crash severity. It is defined by the difference between the vehicle impact speed and the separation speed. The impact speed is the vehicle speed at the crash moment, and the separation speed is calculated based on the conservation of momentum. Then based on the Delta-V we can obtain the occupant injury level. More information can be found in Supplementary Section 1c.

The post-encroachment time (PET) is a widely used surrogate safety measure for characterizing near-miss events. It is defined by the time difference between a vehicle leaving the potential conflict area and a conflicting vehicle entering the same area. We will only consider the PET within the roundabout circle where most conflicts happen. We rasterize the roundabout into $1.3\times 1.3$ meters blocks, and each block is a potential conflict area.

We compare the statistics between the simulated results and the empirical ground-truth data. To quantitatively measure the divergence between two distributions, Hellinger distance and KL-divergence are used as measurements. For two discrete probability distributions ${{{{{\bf{P}}}}}}$ and ${{{{{\bf{Q}}}}}}$, their Hellinger distance ${D}_{H}$ is calculated as follows:

1 $${D}_{H}\left({{{{{\bf{P}}}}}},\,{{{{{\bf{Q}}}}}}\right)=\frac{1}{\sqrt{2}}\sqrt{\mathop{\sum}\limits_{x}{\left(\sqrt{P\left(x\right)}-\sqrt{Q\left(x\right)}\right)}^{2},}$$

which is directly related to the Euclidean norm of the difference between the square root of the two probability vectors. The range of Hellinger distance is between 0 to 1, and the smaller the value, the more similar the two distributions. Suppose ${{{{{\bf{P}}}}}}$ is the real-world data distribution and ${{{{{\bf{Q}}}}}}$ is the simulated distribution, the KL-divergence ${D}_{{KL}}$ can be calculated as

2 $${D}_{{KL}}\left({{{{{\bf{P}}}}}},{{{{{\bf{Q}}}}}}\right)=\mathop{\sum}\limits_{x}P\left(x\right){{\log }}\frac{P\left(x\right)}{Q\left(x\right)}.$$

KL-divergence ranges from 0 to infinity, and also the smaller the value, the more similar the two distributions.

We compare the proposed method with SUMO^17^—a widely used simulation platform for traffic environments, and other state-of-the-art methods. More details for the SUMO simulator settings can be found in Supplementary Section 1d.

### Statistical realism of normal driving behavior

**Fig. 4:**
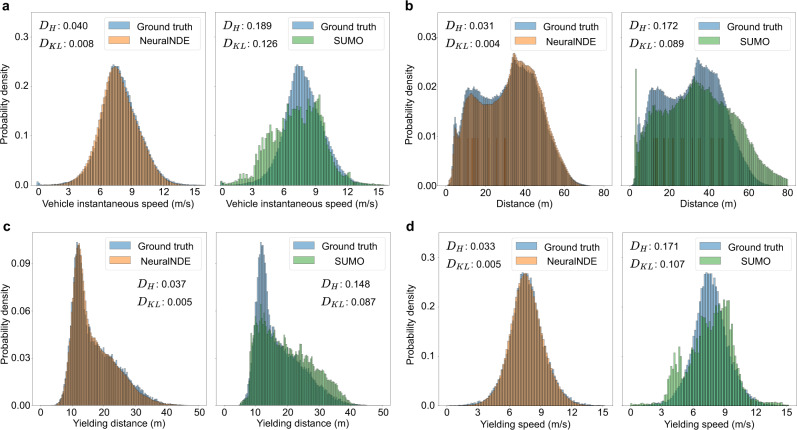
Statistical realism of normal driving behavior. **a** Vehicle instantaneous speed distribution. **b** Vehicle distance distribution. **c** Yielding distance distribution: distance between the yielding vehicle and its nearest conflicting vehicle. **d** Yielding speed distribution: speed of the nearest conflicting vehicle. ${D}_{H}$ and ${D}_{{KL}}$ denote the Hellinger distance and the KL-divergence, respectively.

Since high-fidelity normal driving behavior is the prerequisite for reproducing accurate safety-critical events, in this section, we will first validate the statistical realism of normal driving statistics of the proposed NeuralNDE. An illustration video is provided in Supplementary Movie 2. Vehicle speed and position are direct outcomes of microscopic driving behaviors, and they are critical for both training and testing the AV. The proposed NeuralNDE can generate accurate vehicle instantaneous speed distribution as in the real world, as shown in Fig. 4a. Compared with the SUMO baseline, vehicle speeds in NeuralNDE are naturally distributed among the whole range, covering both low and high-speed situations. Furthermore, NeuralNDE can also accurately reproduce vehicle distance distribution as shown in Fig. 4b, reflecting the full distribution of encounters that AV might face in the real world.

In a two-lane roundabout environment, a highly interactive location is at the roundabout entrance where entering vehicles need to yield to conflicting vehicles within the roundabout. Many real-world conflicts and crashes occur in this location, and the fidelity of these safety-critical events depends on the accuracy of the yielding behavior. Therefore, we will examine the yielding behavior simulated by NeuralNDE to further demonstrate its fidelity in modeling human interactions. The yielding behavior depends on the distance to the conflicting vehicle and the speed of the conflicting vehicle that is traveling within the roundabout. The results of yielding distance and yielding speed distributions are shown in Fig. 4c, d, respectively. We can find that NeuralNDE can accurately replicate human-yielding behavior and significantly outperforms the SUMO simulator. Human drivers are naturally heterogeneous and have different characteristics. Different drivers often exhibit diverse driving behaviors and make different decisions, for example, some drivers are more aggressive and only give way when conflicting vehicles are very close while others might be more conservative. The proposed NeuralNDE is directly learned from real-world data without hand-crafted rules, therefore, it can master the nuanced yielding behavior of human drivers and generate a realistic and diverse driving environment.

### Statistical realism of safety-critical driving behavior

The key challenge of current AV development is how to handle safety-critical driving situations occurring in the real world, therefore, the simulation environment must be able to reproduce these long-tail rare events with high fidelity. In this section, we will examine the performance of NeuralNDE in generating safety-critical events, which include both crash and near-miss situations. The first important statistic is the crash rate. The ground-truth crash rate of the studied roundabout is $1.21\times {10}^{-4}$ crash/km (more details can be found in Supplementary Section 1c). The crash rate of the NeuralNDE is $1.25\times {10}^{-4}$ crash/km, which can accurately reproduce the real-world ground truth.

**Fig. 5:**
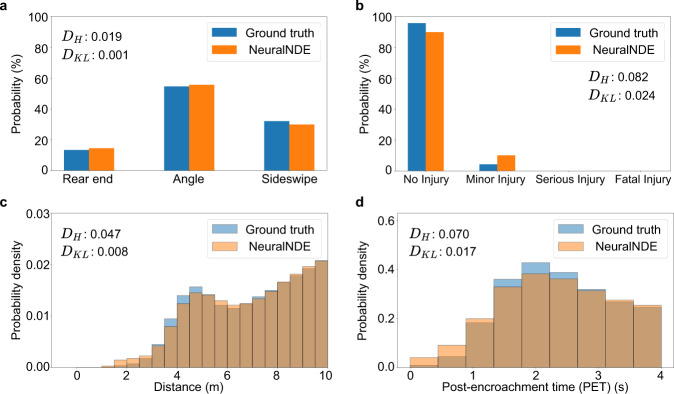
Statistical realism of safety-critical driving behavior. **a** Vehicle crash type distribution. **b** Vehicle crash severity distribution. **c** Vehicle distance distribution in near-miss situations. **d** Post-encroachment time (PET) distribution in near-miss situations. ${D}_{H}$ and ${D}_{{KL}}$ denote the Hellinger distance and the KL-divergence, respectively.

Not only can the proposed NeuralNDE reproduce an accurate crash rate, but also the detailed composition of crash types and crash severity distribution as shown in Fig. 5a and Fig. 5b, respectively. The ground-truth crash type and crash severity distributions are summarized from all crash events that happened at this roundabout from 2016 to 2020 based on police crash reports^50^ (more details can be found in Supplementary Section 1c). These demonstrate that NeuralNDE can generate accurate and diverse crash events following real-world occurring patterns, which are crucial for comprehensive testing of AV performance in different potential crashes. Compared with most state-of-the-art methods, for example, refs. ^30,33–35,51^, none of them compared their simulation results (e.g., crash rates/types/severities) against the real-world data. To the best of our knowledge, we are the only study that validated the simulated safety-critical statistics with real-world ground truth. For each crash type, we will further compare NeuralNDE-generated and real-world crash events in the later section to qualitatively demonstrate the fidelity of our approach. These results validate the capability and effectiveness of the proposed NeuralNDE in generating accurate crash statistics, which is critical for AV applications.

In addition to crashes, near-miss situations are also important. Two measurements, vehicle distance, and PET distributions, are examined to validate the NeuralNDE fidelity. The closest distance between vehicles objectively characterizes potential conflicts between them. To validate the near-miss fidelity, we will focus on the vehicle distance that is smaller than a certain threshold, for example, 10 meters is used in this case. The PET is a widely used surrogate safety measure for identifying near-miss situations. The closer the distance and the smaller the PET, the more dangerous the situation. The results of the distance distribution in near-miss situations are shown in Fig. 5c. We can find that NeuralNDE can replicate the distance in near-miss situations with high accuracy. Similarly, the simulated PET distribution can also accurately reproduce real-world dangerous driving conditions as shown in Fig. 5d. These results demonstrate that in addition to crashes, NeuralNDE can also characterize real-world near-miss statistics, which validates the modeling accuracy of the proposed method regarding vehicle safety-critical behaviors.

To validate the effectiveness of each module of the proposed framework, we conducted ablation studies and the result can be found in Supplementary Section 2a. It demonstrates that the Transformer-based behavior modeling network and the generative adversarial training technique are important for modeling multi-agent interaction behavior, and conflict critic module and safety mapping network are essential for generating realistic safety-critical events.

### Generated crash events

**Fig. 6:**
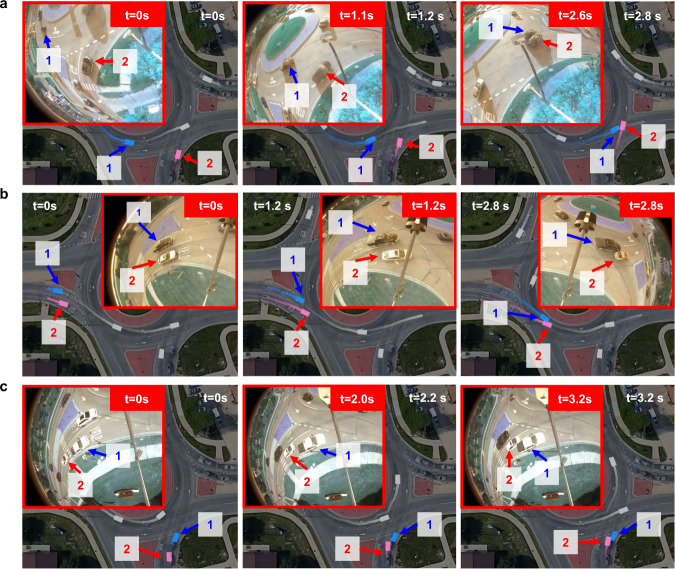
Crash events in the real world and NeuralNDE. Each subfigure denotes a different crash type. The main image in each subfigure demonstrates the crash event generated by NeuralNDE. The image in the red box denotes the real-world crash event captured by roadside cameras. **a** Angle crash caused by failure to yield. Vehicle #2 (shown in pink) fails to yield to vehicle #1 (shown in blue) and causes the collision. **b** Sideswipe crash caused by improper lane usage. One vehicle improperly intrudes into the lane of another vehicle and causes the collision. **c** Rear-end crash caused by failure to stop within assured clear distance. Vehicle #2 (shown in pink) fails to keep a safe distance from vehicle #1 (shown in blue) and causes the collision.

The proposed NeuralNDE can generate complex and diverse interactions that happen in real-world traffic. Human drivers naturally exhibit different characteristics and spontaneously interact, negotiate, and cooperate to navigate through the roundabout, as shown in Supplementary Movie 1. During vehicle interactions, crashes may happen due to different reasons, for example, failure to yield, improper lane usage, etc. In this section, we showcase three generated crashes by NeuralNDE. By comparing them with real-world crash events, we can demonstrate that NeuralNDE can generate realistic and diverse crash patterns. These results further validate NeuralNDE fidelity on vehicle safety-critical behaviors which are very difficult to model. The illustration figures of the three crash examples with corresponding real-world crash events are shown in Fig. 6. The video version of these events can be found in Supplementary Movie 3.

The first case is an angle crash caused by failure to yield as shown in Fig. 6a, where the main image denotes the crash event generated by NeuralNDE, and the image in the red box is a real-world crash event. For the NeuralNDE results, vehicles’ current states and their past trajectories are shown by rectangles and lines, respectively. For better visualization, only vehicles that are of our interest are shown in colors and other vehicles are shown in gray. In this case, vehicle #1 (shown in blue) is circulating within the roundabout, and vehicle #2 (shown in pink) is at the south entrance. We can find that vehicle #2 fails to yield to the right-of-way of vehicle #1, and chooses to enter the roundabout aggressively. As a result, vehicle #1 cannot decelerate in time and a crash happens. The generated crash is very similar to what would happen in the real world as shown by the images in the red box of Fig. 6a. As captured by the roadside camera, vehicle #2 at the entrance fails to yield and finally crashed with vehicle #1 within the roundabout.

The second case is a sideswipe crash caused by improper lane usage as shown in Fig. 6b. In this case, two vehicles enter the roundabout from the west entrance side by side. Vehicle #1 (shown in blue) drives in the inner lane and vehicle #2 (shown in pink) drives in the outer lane. When they are approaching the south part of the roundabout, vehicle #2 recklessly steers into vehicle #1’s lane and leads to a crash. This type of improper lane usage crash also frequently occurs in the real world. As shown by the images in the red box of Fig. 6b, vehicle #1 also improperly intrude into the lane of vehicle #2, causing a crash to happen.

The third case is a rear-end crash caused by failure to stop within assured clear distance. In this case, vehicle #1 (shown in blue) is stopped and waiting to enter the roundabout, while vehicle #2 (shown in pink) fails to maintain a safe distance from vehicle #1 and causes a rear-end collision. The NeuralNDE-generated crash is very similar to the crash event happening in real traffic as shown in Fig. 6c. From these results, we can find that NeuralNDE can generate realistic crash events that occur in the real world. The ability to reproduce these rare safety-critical events is essential for AV testing.

### Model scalability

Modeling a large traffic network is more challenging than modeling individual scenarios because of two reasons: 1. It will be difficult to obtain full trajectory data for all vehicles in the network; 2. Error accumulation issue may become more noticeable because the elapsed time for each agent will be longer. The key idea for extending to a traffic network is that a large network can be decomposed into subareas, where critical subareas (e.g., intersection, roundabout, highway entrance and exit, etc.) that involve complex interactions will be controlled by NeuralNDE models, and other subareas (e.g., road segments connecting different scenarios, etc.) can be controlled by traditional rule-based models. Therefore, we only need to have trajectory data to build NeuralNDE models for those critical nodes in a large network, and connect these nodes with links that are modeled by traditional rule-based approaches (for example, car-following and lane-changing models).

**Fig. 7:**
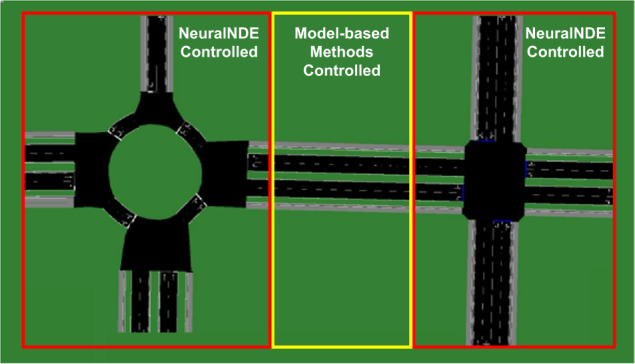
Proof-of-concept for modeling a road network. It involves two scenarios, i.e., a four-way intersection and a two-lane roundabout, The areas denoted by red rectangles are controlled by NeuralNDE methods, and the areas denoted by yellow rectangles are controlled by model-based methods.

As a proof-of-concept, we build a “network”, as shown in Fig. 7, that involves two scenarios, i.e., a four-way stop sign-controlled intersection and a two-lane roundabout. We use SUMO^17^ simulator to generate vehicle trajectory data and use it as the ground truth of the NDE. We assume the traffic network is not fully perceptional and we only have vehicle trajectory data in the intersection and roundabout areas. Therefore, these two areas are controlled by trained NeuralNDE models, and the transition areas between the two scenarios are controlled by rule-based IDM car-following model^13^ and SL2015 lane-changing model^17^. The proposed method can generate high-fidelity simulation, as shown in Supplementary Movie 4. More details about experiment settings can be found in Supplementary Section 1f.

**Fig. 8:**
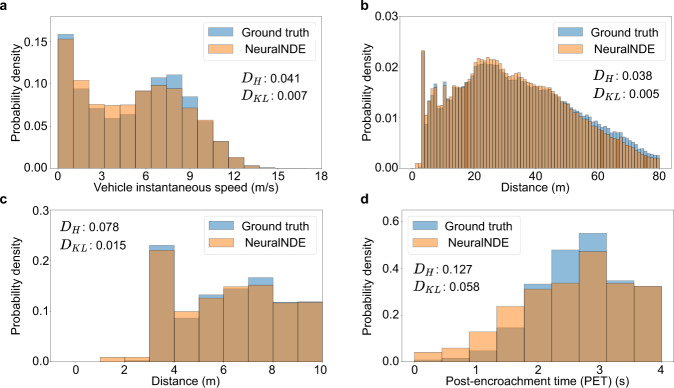
Statistical realism of the intersection area in the road network. **a** Vehicle instantaneous speed distribution. **b** Vehicle distance distribution. **c** Vehicle distance distribution in near-miss situations. **d** Post-encroachment time (PET) distribution in near-miss situations. ${D}_{H}$ and ${D}_{{KL}}$ denote the Hellinger distance and the KL-divergence, respectively.

We simulate the network and collect the data in the intersection and roundabout areas to quantitively evaluate the performance. The simulated network can still achieve statistical realism and the results are discussed below. We run around 100 hours of simulation to collect the data. For the intersection area, we evaluate vehicle instantaneous speed and distance distributions to demonstrate the performance of normal driving behavior, as shown in Fig. 8a, b, respectively. From the results, we can find that the simulated distribution is consistent with the ground truth. We further validate the statistical realism of the safety-critical driving behavior. The results of vehicle distance in near-miss situations (smaller than 10 meters) and PET are shown in Fig. 8c, d, respectively. We can find that the proposed method can replicate the ground-truth with high accuracy.

**Fig. 9:**
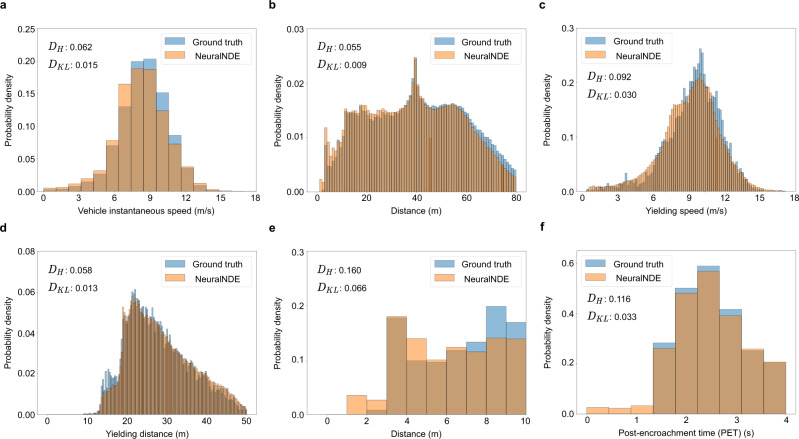
Statistical realism of the roundabout area in the road network. **a** Vehicle instantaneous speed distribution. **b** Vehicle distance distribution. **c** Yielding speed distribution. **d** Yielding distance distribution. **e** Vehicle distance distribution in near-miss situations. **f** Post-encroachment time (PET) distribution in near-miss situations. ${D}_{H}$ and ${D}_{{KL}}$ denote the Hellinger distance and the KL-divergence, respectively.

For the roundabout area, the vehicle instantaneous speed, distance, yielding speed, and yielding distance results are shown in Fig. 9a–d, respectively. The safety-critical events metrics (vehicle distance in the near-miss situations and PET) are shown in Fig. 9e, f, which also demonstrate satisfactory performance. These results serve as a proof-of-concept to demonstrate the performance and scalability potential of our proposed NeuralNDE models for simulating large traffic networks.

## Discussion

The proposed NeuralNDE demonstrates promising performance for modeling a complex urban driving environment with statistical realism for both normal and safety-critical driving conditions. To the best of the authors’ knowledge, this is the first time that a simulation environment can be statistically representative of the real-world driving environment. More importantly, it can accurately characterize long-tail rare-event statistics, for example, crash rate, crash type, and crash severity distributions, which are very difficult to achieve but will notably influence AV training and testing accuracy.

For simulation-based AV testing, there are two critical problems that need to be solved. The first one is how to build a high-fidelity simulation environment and the second one is how to develop an accelerated testing methodology that can evaluate the AV performance accurately and efficiently. This work is focusing on the first problem, where the high-fidelity simulator is a prerequisite and foundation for simulation-based AV testing applications^2,4,52^. In our prior works^2,4^, we have developed accelerated testing methodologies that use a purely data-driven NDE model, which can be replaced with the high-fidelity NeuralNDE. We should note that human-driven vehicles might behave differently if they are interacting with an AV^53^. Therefore, further development and enhancement for NeuralNDE might be required in the future to consider the AV influences on surrounding vehicles.

## Methods

### Behavior modeling network

We frame the behavior modeling via imitation learning with the help of large-scale real-world offline demonstrations. Given a large-scale collection of real-world vehicle trajectory data, we aim to jointly model both vehicle-to-vehicle interactions and their long-term state trajectories within a certain temporal range. In our framework, we consider each vehicle instance as an agent with stochastic actions and future states where the actions and states of each agent are not only related to its own historical trajectories, but also to that of all other agents.

Suppose ${{{{{{\bf{s}}}}}}}_{{{{{{\bf{i}}}}}}}^{{{{{{\bf{t}}}}}}}$ and ${{{{{{\bf{a}}}}}}}_{{{{{{\bf{i}}}}}}}^{{{{{{\bf{t}}}}}}}$ represent the state vector (e.g., location, pose, vehicle size, etc.) and action vector (e.g., acceleration, yaw rate, etc.) of $i$th agent at timestep $t$. ${{{{{{\bf{S}}}}}}}_{{{{{{\bf{N}}}}}}}^{{{{{{\boldsymbol{\tau }}}}}}{{{{{\boldsymbol{:}}}}}}{{{{{\bf{t}}}}}}}=\{{{{{{{\bf{s}}}}}}}_{{{{{{\bf{1}}}}}}}^{{{{{{\bf{t}}}}}}{{{{{\boldsymbol{-}}}}}}{{{{{\boldsymbol{\tau }}}}}}{{{{{\boldsymbol{+}}}}}}{{{{{\bf{1}}}}}}},\ldots {{{{{{\bf{s}}}}}}}_{{{{{{\bf{1}}}}}}}^{{{{{{\bf{t}}}}}}},\ldots {{{{{{\bf{s}}}}}}}_{{{{{{\bf{N}}}}}}}^{{{{{{\bf{t}}}}}}{{{{{\boldsymbol{-}}}}}}{{{{{\boldsymbol{\tau }}}}}}{{{{{\boldsymbol{+}}}}}}{{{{{\bf{1}}}}}}},\ldots {{{{{{\bf{s}}}}}}}_{{{{{{\bf{N}}}}}}}^{{{{{{\bf{t}}}}}}}\}$ represents a collection of the state trajectories of all $N$ agents from all $\tau$ timesteps ahead of the current time $t$. The modeling of all agents’ future actions can be thus essentially considered as a conditional probabilistic inference problem, i.e., to estimate the joint distribution of actions from all agents $p({{{{{{\bf{a}}}}}}}_{{{{{{\bf{1}}}}}}}^{{{{{{\bf{t}}}}}}},\ldots,{{{{{{\bf{a}}}}}}}_{{{{{{\bf{N}}}}}}}^{{{{{{\bf{t}}}}}}}|{{{{{{\bf{S}}}}}}}_{{{{{{\bf{N}}}}}}}^{{{{{{\boldsymbol{\tau }}}}}}{{{{{\boldsymbol{:}}}}}}{{{{{\bf{t}}}}}}})$ given their historical states as conditional inputs. To accurately model the joint distribution, the Transformer model is used as the backbone of our behavior modeling network. Transformer models originated from the field of natural language processing^54^, and have revealed remarkable performance in many applications, including computer vision^55^, bioinformatics^56^, and multimodal data generative modeling^57^.

There are three advantages to modeling each agent as a “token” in the language model. The first advantage is that the Transformer is naturally suitable for modeling long-term interactive behavior in a multi-agent environment. The self-attention mechanism is capable of characterizing inter-token relations, which model the interaction between agents. The position-wise feed-forward network in the Transformer can capture intra-token information, which measures the influence of the historical states of each agent on their future behavior. The second advantage is model scalability. In this study, our model can handle up to 32 objects simultaneously, considering the size of the roundabout and for the convenience of experiments. However, the framework we designed should be able to handle a much larger number of objects, as each agent in the simulation is modeled as a “token”. It has been shown in previous studies^58,59^ that the Transformer can handle up to thousands of “tokens”. The third advantage is the permutation invariant property. The Transformer block is permutation invariant to the order of tokens. Therefore, by modeling each agent as a token, we do not need to specify the order of agents, which are geographically located in a two-dimensional space (i.e., on a road), making it difficult to order them in a one-dimensional space (i.e., determine the token order in input).

At each timestep of modeling, the behavior modeling network ${{{{{{\rm{F}}}}}}}_{{{{{{\rm{M}}}}}}}$ takes in the historical states ${{{{{{\bf{S}}}}}}}_{{{{{{\bf{N}}}}}}}^{{{{{{\boldsymbol{\tau }}}}}}{{{{{\boldsymbol{:}}}}}}{{{{{\bf{t}}}}}}}$ of all agents and is trained to jointly predict their future actions (${{{{{{\bf{a}}}}}}}_{{{{{{\bf{1}}}}}}}^{{{{{{\bf{t}}}}}}},\ldots,{{{{{{\bf{a}}}}}}}_{{{{{{\bf{N}}}}}}}^{{{{{{\bf{t}}}}}}}$). Instead of predicting deterministic actions values, we predict the stepwise action distributions and consider distribution as a multi-variable Gaussian over their action space:

3 $$p\left({{{{{{\bf{S}}}}}}}_{{{{{{\bf{N}}}}}}}^{{{{{{\boldsymbol{\tau }}}}}}:{{{{{\bf{t}}}}}}}\right)={{{{{{\rm{F}}}}}}}_{{{{{{\rm{M}}}}}}}\left({{{{{{\bf{S}}}}}}}_{{{{{{\bf{N}}}}}}}^{{{{{{\boldsymbol{\tau }}}}}}:{{{{{\bf{t}}}}}}}\right) \sim N\left({{{{{{\mathbf{\mu }}}}}}}_{{{{{{\bf{a}}}}}},{{{{{\bf{i}}}}}}={{{{{\bf{1}}}}}}\ldots {{{{{\bf{N}}}}}}}^{{{{{{\bf{t}}}}}}},{{{{{{\mathbf{\Sigma }}}}}}}_{{{{{{\bf{a}}}}}},{{{{{\bf{i}}}}}}={{{{{\bf{1}}}}}}\ldots {{{{{\bf{N}}}}}}}^{{{{{{\bf{t}}}}}}}\right),$$

where $p\left({{{{{{\bf{S}}}}}}}_{{{{{{\bf{N}}}}}}}^{{{{{{\boldsymbol{\tau }}}}}}{{{{{\boldsymbol{:}}}}}}{{{{{\bf{t}}}}}}}\right)$ is the joint action distribution and ${{{{{{\mathbf{\mu }}}}}}}_{{{{{{\bf{a}}}}}}{{{{{\boldsymbol{,}}}}}}{{{{{\bf{i}}}}}}{{{{{\boldsymbol{=}}}}}}{{{{{\bf{1}}}}}}{{{{{\boldsymbol{\ldots }}}}}}{{{{{\bf{N}}}}}}}^{{{{{{\bf{t}}}}}}}$ and ${{{{{{\mathbf{\Sigma }}}}}}}_{{{{{{\bf{a}}}}}}{{{{{\boldsymbol{,}}}}}}{{{{{\bf{i}}}}}}{{{{{\boldsymbol{=}}}}}}{{{{{\bf{1}}}}}}{{{{{\boldsymbol{\ldots }}}}}}{{{{{\bf{N}}}}}}}^{{{{{{\bf{t}}}}}}}$ are the mean and covariance matrix of the Gaussian distribution. After we obtain the joint distribution of actions, a group of action vectors for each agent are sampled:

4 $${{{{{{\bf{a}}}}}}}_{{{{{{\bf{1}}}}}}}^{{{{{{\bf{t}}}}}}},\ldots,{{{{{{\bf{a}}}}}}}_{{{{{{\bf{N}}}}}}}^{{{{{{\bf{t}}}}}}}\leftarrow N\left({{{{{{\mathbf{\mu }}}}}}}_{{{{{{\bf{a}}}}}},{{{{{\bf{i}}}}}}={{{{{\bf{1}}}}}} \ldots {{{{{\bf{N}}}}}}}^{{{{{{\bf{t}}}}}}},{{{{{{\mathbf{\Sigma }}}}}}}_{{{{{{\bf{a}}}}}},{{{{{\bf{i}}}}}}={{{{{\bf{1}}}}}} \ldots {{{{{\bf{N}}}}}}}^{{{{{{\bf{t}}}}}}}\right).$$

Then, for each agent, its new state vector ${{{{{{\bf{s}}}}}}}_{{{{{{\bf{i}}}}}}}^{{{{{{\bf{t}}}}}}{{{{{\boldsymbol{+}}}}}}{{{{{\bf{1}}}}}}}$ is determined by a differentiable state transition function ${{{{{\rm{T}}}}}}$ determined by vehicle dynamics:

5 $${{{{{{\bf{s}}}}}}}_{{{{{{\bf{i}}}}}}}^{{{{{{\bf{t}}}}}}+{{{{{\bf{1}}}}}}}={{{{{\rm{T}}}}}}\left({{{{{{\bf{a}}}}}}}_{{{{{{\bf{i}}}}}}}^{{{{{{\bf{t}}}}}}},{{{{{{\bf{s}}}}}}}_{{{{{{\bf{i}}}}}}}^{{{{{{\bf{t}}}}}}}\right).$$

The above processing will be repeated so that new states of all agents can be generated in an auto-regressive manner. In practice, instead of one-step prediction, multiple timesteps (e.g., $\kappa$ steps) predictions ${{{{{{\bf{S}}}}}}}_{{{{{{\bf{N}}}}}}}^{{{{{{\bf{t}}}}}}{{{{{\boldsymbol{:}}}}}}{{{{{\boldsymbol{\kappa }}}}}}}=\{{{{{{{\bf{s}}}}}}}_{{{{{{\bf{1}}}}}}}^{{{{{{\bf{t}}}}}}+{{{{{\bf{1}}}}}}},\ldots {{{{{{\bf{s}}}}}}}_{{{{{{\bf{1}}}}}}}^{{{{{{\bf{t}}}}}}+{{{{{\boldsymbol{\kappa }}}}}}},\ldots {{{{{{\bf{s}}}}}}}_{{{{{{\bf{N}}}}}}}^{{{{{{\bf{t}}}}}}+{{{{{\bf{1}}}}}}},\ldots {{{{{{\bf{s}}}}}}}_{{{{{{\bf{N}}}}}}}^{{{{{{\bf{t}}}}}}+{{{{{\boldsymbol{\kappa }}}}}}}\}$ will be made by the behavior modeling network. Note that to simulate the uncertainty of drivers, during the simulation, at each timestep, we will sample from the joint distribution to determine all agents’ future trajectories and then simulate forward. Also, our model can be easily extended to generate multimodal outputs, where several Gaussian distributions instead of one will be predicted to further improve the uncertainty of drivers^60^.

**Fig. 10:**
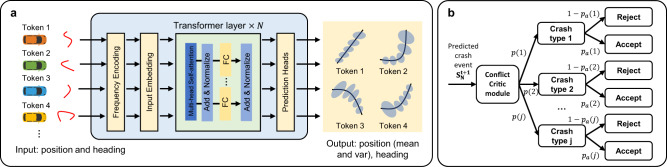
Illustration of individual modules. **a** Network architecture of the behavior modeling network. **b** The illustration of the conflict critic module.

The proposed behavior modeling network consists of a frequency encoding layer, an input embedding layer, a Transformer backbone, and a prediction layer, as shown in Fig. 10a. Detailed network architecture can be found in the later section. The input embedding layer is a fully connected layer with weights shared across different tokens. The Transformer backbone consists of several standard BERT^41^ layers stacked on top of each other. Since the action prediction is independent of the input order of the $N$ agent, we, therefore, have removed the “positional encoding”, which is a standard encoding layer in Transformers to capture order-related information for sequence input data. Also, before the state vectors are input to the input embedding layer, we adopt the ideas of Mildenhall et al. ^61^ to use frequency encoding, which applies a set of sine and cosine basis functions that projects the vectors to high-dimensional space to improve capturing high-frequency variation in the state spaces. Suppose $\gamma$ defines a mapping function from ${R}^{1}$ to ${R}^{2L+1}$, where $L$ is the order of frequencies. The state value ${{{{{\bf{s}}}}}}$ after mapping can be written as follows:

6 $${{{{{\boldsymbol{\gamma }}}}}}\left({{{{{\boldsymbol{s}}}}}}\right)=\left[{{{{{\bf{s}}}}}},{{\sin }}\left({2}^{0}\pi {{{{{\bf{s}}}}}}\right),{{\cos }}\left({2}^{0}\pi {{{{{\bf{s}}}}}}\right),\ldots,{{\sin }}\left({2}^{L-1}\pi {{{{{\bf{s}}}}}}\right),{{\cos }}\left({2}^{L-1}\pi {{{{{\bf{s}}}}}}\right)\right].$$

To train the model ${{{{{{\rm{F}}}}}}}_{{{{{{\rm{M}}}}}}}$ with implicit variance, in the prediction layer, two prediction heads are attached for each input token at the output end, one for predicting ${{{{{{\mathbf{\mu }}}}}}}_{{{{{{\bf{a}}}}}}{{{{{\boldsymbol{,}}}}}}{{{{{\bf{i}}}}}}}^{{{{{{\bf{t}}}}}}}$, another for predicting ${{{{{{\mathbf{\Sigma }}}}}}}_{{{{{{\bf{a}}}}}}{{{{{\boldsymbol{,}}}}}}{{{{{\bf{i}}}}}}}^{{{{{{\bf{t}}}}}}}$. The training of the behavior modeling network can be formulated as a maximum likelihood estimation process. Given $N$ agents of $T$ timesteps, the state trajectories within [$t-\tau$, $t$] are used as input and the action vectors at time $t$ are used as the ground truth ($t=1,\ldots,T$), then the likelihood function can be written as follows:

7 $$p\left({{{{{{\rm{F}}}}}}}_{{{{{{\rm{M}}}}}}}\right)=p\left({{{{{{\bf{s}}}}}}}_{{{{{{\bf{1}}}}}}}^{{{{{{\bf{1}}}}}}},{{{{{{\bf{s}}}}}}}_{{{{{{\bf{1}}}}}}}^{{{{{{\bf{2}}}}}}},\ldots,\,{{{{{{\bf{s}}}}}}}_{{{{{{\bf{i}}}}}}}^{{{{{{\bf{t}}}}}}},\ldots,{{{{{{\bf{s}}}}}}}_{{{{{{\bf{N}}}}}}}^{{{{{{\bf{T}}}}}}}\right).$$

For simplification, we assume that there is no correlation between variables in the multivariate Gaussian distribution, so the action covariance matrix for each agent is a diagonal matrix, i.e., ${{{{{{\boldsymbol{\Sigma }}}}}}}_{{{{{{\bf{a}}}}}}{{{{{\boldsymbol{,}}}}}}{{{{{\bf{i}}}}}}}^{{{{{{\bf{t}}}}}}} \, \approx \, {diag}({\sigma }_{i,1}^{t},\ldots,{\sigma }_{i,D}^{t})$, $D$ is the dimension of the action vector. Then the joint probability of an action vector ${{{{{{\bf{a}}}}}}}_{{{{{{\bf{i}}}}}}}^{{{{{{\bf{t}}}}}}}$ can be implied as follows:

8 $$p\left({{{{{{\bf{s}}}}}}}_{{{{{{\bf{i}}}}}}}^{{{{{{\bf{t}}}}}}}\right)=\mathop{\prod}\limits_{j=1}^{D}\frac{1}{{\left(2\pi \right)}^{\frac{1}{2}}}\frac{1}{{\sigma }_{i,j}^{t}}\exp \left\{ - \frac{{\left({\mu }_{i,j}^{t}-{a}_{i,j}^{t}\right)}^{2}}{2{({\sigma }_{i,j}^{t})}^{2}}\right\},$$

where ${\mu}_{i,j}^{t}$ represents the predicted $j$th action value at time $t$ for $i$th agent.

We approximate the joint probability distribution $p ({{{{{\rm{F}}}}}}_{{{{{\rm{M}}}}}})$ in Eq. (7) as the multiplicative form of each agent’s marginal probabilities, and combine it with Eq. (8) to derive the loss function in the negative log-likelihood form as follows:

9 $${{{{{{\rm{L}}}}}}}_{{{{{{\rm{M}}}}}}}\left({{{{{{\rm{F}}}}}}}_{{{{{{\rm{M}}}}}}}\right)=\mathop{\sum }\limits_{t=1}^{T}\mathop{\sum }\limits_{i=1}^{N}\mathop{\sum }\limits_{j=1}^{D}\left[{{{{{\rm{ln}}}}}}\left({\sigma }_{i,j}^{t}\right)+\frac{{\left({\mu }_{i,j}^{t}-{a}_{i,j}^{t}\right)}^{2}}{2{\left({\sigma }_{i,j}^{t}\right)}^{2}}\right].$$

The approximation of Eq. (7) will not affect the solution since the term $\frac{1}{2}{({\mu }_{i,j}^{t}-{a}_{i,j}^{t})}^{2}/{({\sigma }_{i,j}^{t})}^{2}$ in Eq. (9), which represents the expected prediction accuracy of the actions, can still make the model converge to the optimal solution. Note that although we don’t have ground truth for the predicted variance ${\sigma }_{i,j}^{t}$, it can be jointly estimated as implicit variables along with the mean action ${\mu }_{i,j}^{t}$ during the training process, where a high uncertainty prediction naturally responds to a large variance and vice versa. The training details can be found in Supplementary Section 1e.

### Conflict critic module

The simulated environment must be able to reproduce accurate safety-critical driving statistics including both near-miss and crash events. Although the behavior modeling network can generate realistic conflicts, it may not be able to achieve distribution-level accuracy, due to the rarity of safety-critical events in the training dataset. For example, the crash rate can be unrealistically high, and the crash type distribution can be inconsistent with the real-world driving environment. To tackle this issue, we design a model-based conflict critic module ${{{{{{\rm{F}}}}}}}_{{{{{{\rm{c}}}}}}}$ to control the occurring frequencies and patterns of safety-critical behaviors during the inference time to achieve statistical realism, as illustrated in Fig. 2b. The input to ${{{{{{\rm{F}}}}}}}_{{{{{{\rm{c}}}}}}}$ are the sampled $\kappa$ steps predicted trajectories of all $N$ agents ${{{{{{\bf{S}}}}}}}_{{{{{{\bf{N}}}}}}}^{{{{{{\bf{t}}}}}}{{{{{\boldsymbol{:}}}}}}{{{{{\boldsymbol{\kappa }}}}}}}=\{{{{{{{\bf{s}}}}}}}_{{{{{{\bf{1}}}}}}}^{{{{{{\bf{t}}}}}}{{{{{\boldsymbol{+}}}}}}{{{{{\bf{1}}}}}}},\ldots {{{{{{\bf{s}}}}}}}_{{{{{{\bf{1}}}}}}}^{{{{{{\bf{t}}}}}}{{{{{\boldsymbol{+}}}}}}{{{{{\boldsymbol{\kappa }}}}}}},\ldots {{{{{{\bf{s}}}}}}}_{{{{{{\bf{N}}}}}}}^{{{{{{\bf{t}}}}}}{{{{{\boldsymbol{+}}}}}}{{{{{\bf{1}}}}}}},\ldots {{{{{{\bf{s}}}}}}}_{{{{{{\bf{N}}}}}}}^{{{{{{\bf{t}}}}}}{{{{{\boldsymbol{+}}}}}}{{{{{\boldsymbol{\kappa }}}}}}}\}$ generated by the behavior modeling network at the current time $t$. The output of ${{{{{{\rm{F}}}}}}}_{{{{{{\rm{c}}}}}}}$ is the acceptance probability ${p}_{a}$ for not passing through the safety mapping network:

10 $${p}_{a}={{{{{{\rm{F}}}}}}}_{{{{{{\rm{c}}}}}}}\left({{{{{{\bf{S}}}}}}}_{{{{{{\bf{N}}}}}}}^{{{{{{\bf{t}}}}}}{{{{{\boldsymbol{:}}}}}}{{{{{\boldsymbol{\kappa }}}}}}}\right).$$

If there is a potential conflict in predicted trajectories ${{{{{{\bf{S}}}}}}}_{{{{{{\bf{N}}}}}}}^{{{{{{\bf{t}}}}}}{{{{{\boldsymbol{:}}}}}}{{{{{\boldsymbol{\kappa }}}}}}},$ we will have a probability ${p}_{a}$ to accept it, and a probability $1-{p}_{a}$ to reject it and let the safety mapping network guide and rectify the dangerous driving behavior. The acceptance probability is trajectory-dependent, which means that for those conflict patterns that have a higher probability of occurring in the real world, we will have correspondingly higher ${p}_{a}$ to accept it. Therefore, by calibrating the ${{{{{{\rm{F}}}}}}}_{{{{{{\rm{c}}}}}}}$ function, we can control the generation process of safety-critical events to match real-world statistics in both near-misses and crashes. Specifically, in the study, each crash type will have a specific acceptance probability. The implementation details and calibration methods are introduced in the following paragraphs.

In this study, we consider vehicle conflicts in a one-step prediction for simplicity. Let ${{{{{{\bf{S}}}}}}}_{{{{{{\bf{N}}}}}}}^{{{{{{\bf{t}}}}}}{{{{{\boldsymbol{+}}}}}}{{{{{\bf{1}}}}}}}$ denotes all vehicle states predicted by the behavior modeling network at the next timestep $t+1$. If there is a crash happening in the predicted trajectory, we will have a certain probability to accept the crash and generate it, otherwise, the vehicle behavior will be rectified by the safety mapping network to avoid the crash. The acceptance probability will depend on the predicted crash type that happens in ${{{{{{\bf{S}}}}}}}_{{{{{{\bf{N}}}}}}}^{{{{{{\bf{t}}}}}}{{{{{\boldsymbol{+}}}}}}{{{{{\bf{1}}}}}}}$ and will be calibrated as discussed in the next paragraph. For the same crash type, the acceptance probability will be the same. If there is no crash in ${{{{{{\bf{S}}}}}}}_{{{{{{\bf{N}}}}}}}^{{{{{{\bf{t}}}}}}{{{{{\boldsymbol{+}}}}}}{{{{{\bf{1}}}}}}}$, the acceptance probability will be zero. An illustration figure is shown in Fig. 10b. By calibrating the conflict critic module, i.e., obtaining the acceptance probability ${p}_{a}\left(j\right)$ for different crash types $j$, we can realize accurate crash rate and crash type distribution of the simulation environment.

The calibration process is divided into two steps, where the first step aims to fit the crash rate and the second step tries to fit the crash type distribution. In the first step, we first assume a uniform acceptance probability (${p}_{{ua}}$) for different crash types and try to fit the ground-truth crash rate. The calibration process is, at the first iteration, making a random initial guess of the uniform acceptance probability ${p}_{{ua}}^{1}\in ({{{{\mathrm{0,1}}}}}]$, then run simulations to obtain the current NeuralNDE crash rate at the first iteration ${c}^{1}$. Then linearly update the uniform acceptance probability as follows

11 $${p}_{{ua}}^{i+1}={c}^{{gt}}\cdot \frac{{p}_{{ua}}^{i}}{{c}^{i}},$$

where ${c}^{{gt}}$ denotes the desired ground-truth crash rate, and $i$ denotes the current iteration number. Continue this process until the NeuralNDE crash rate is close to the ground truth with satisfactory accuracy. In the second step, we will calibrate the acceptance probability for each crash type. The acceptance probability ${p}_{a}\left(j\right)$ for crash type $j$ needs to satisfy the following system of linear equations to fit both crash rate (Eq. (12)) and crash type distribution (Eq. (13)):

12 $$\mathop{\sum }_{j}p\left(j\right){p}_{a}\left(j\right) \,=\, {p}_{{ua}}.$$

13 $$\frac{p\left(j\right){p}_{a}\left(j\right)}{{\sum }_{j}p\left(j\right){p}_{a}\left(j\right)} \,=\, {c}^{{gt}}\left(j\right),\forall j\in J,$$

where ${c}^{{gt}}\left(j\right)$ is the ground-truth probability of crash type $j$, ${p}_{{ua}}$ is the uniform acceptance probability obtained from the first step, and $p\left(j\right)$ is the probability of crash type $j$ occurring in NeuralNDE using the uniform probability ${p}_{{ua}}$. For Eq. (12), the summation of $p\left(j\right){p}_{a}\left(j\right)$ overall potential crash types $j$ is the overall acceptance probability considering different crash types. It needs to be equal to the uniform acceptance probability (${p}_{{ua}}$) obtained in the first step, which can guarantee the accurate crash rate of the simulation. Therefore, the acceptance probability ${p}_{a}\left(j\right)$ equals to

14 $${p}_{a}\left(j\right)={p}_{{ua}}\cdot \frac{{c}^{{gt}}\left(j\right)}{p\left(j\right)}.$$

We will use ${p}_{a}\left(j\right)$ as the acceptance probability of different crash types $j\in J$ for the conflict critic module.

### Safety mapping network

To improve the modeling accuracy and achieve statistical realism in safety-critical conditions, we propose a safety mapping network that can guide vehicle behavior in safety-critical situations by mapping the unsafe vehicle behaviors to their closest safe neighbors. The safety mapping network serves as a safety guard to rectify vehicle behaviors before an imminent crash. Given the current state and predicted $\kappa$ steps future actions of all agents $\{{{{{{{\bf{S}}}}}}}_{{{{{{\bf{N}}}}}}}^{{{{{{\bf{t}}}}}}},\,{{{{{{\bf{A}}}}}}}_{{{{{{\bf{N}}}}}}}^{{{{{{\bf{t}}}}}}{{{{{\boldsymbol{:}}}}}}{{{{{\boldsymbol{\kappa }}}}}}}\}$, the safety mapping network ${{{{{{\rm{F}}}}}}}_{{{{{{\rm{S}}}}}}}$ jointly predicts the rectified actions ${{{{{{\bf{A}}}}}}}_{{{{{{\bf{N}}}}}}}^{{{{{{\bf{t}}}}}}{{{{{\boldsymbol{:}}}}}}{{{{{\boldsymbol{\kappa }}}}}},{{{{{\boldsymbol{*}}}}}}}$ of all agents as follows

15 $${{{{{{\bf{A}}}}}}}_{{{{{{\bf{N}}}}}}}^{{{{{{\bf{t}}}}}}:{{{{{\boldsymbol{\kappa }}}}}},*}={{{{{{\rm{F}}}}}}}_{{{{{{\rm{S}}}}}}}\left({{{{{{\bf{S}}}}}}}_{{{{{{\bf{N}}}}}}}^{{{{{{\bf{t}}}}}}},\,{{{{{{\bf{A}}}}}}}_{{{{{{\bf{N}}}}}}}^{{{{{{\bf{t}}}}}}:{{{{{\boldsymbol{\kappa }}}}}}}\right).$$

If there is an impending crash using the original action vector ${{{{{{\bf{A}}}}}}}_{{{{{{\bf{N}}}}}}}^{{{{{{\bf{t}}}}}}{{{{{\boldsymbol{:}}}}}}{{{{{\boldsymbol{\kappa }}}}}}}$, the safety mapping network will modify the action vector to resolve the potential conflict. Note that the action rectification will only be done if the original action vector will result in a predicted crash, otherwise the action output by the safety mapping network will be the same as the original action vector.

The safety mapping network is trained to imitate existing model-based safety guards based on domain knowledge. In this study, for simplicity and generality, we consider one-step prediction and use a physics-based safety guard as the training target. The illustration figure is shown in Supplementary Fig. 2. When two vehicles are going to collide with each other, we resolve the potential conflict by setting a repulsive force between them. The force is projected to the heading direction of each vehicle and restricts their action to avoid the crash. We generate a large number of offline random states-response pairs based on the above rules. The loss function for training the safety mapping network can be written as follows:

16 $${{{{{{\rm{L}}}}}}}_{{{{{{\rm{S}}}}}}}\left({{{{{{\rm{F}}}}}}}_{{{{{{\rm{S}}}}}}}\right)={{||}{{{{{{\rm{F}}}}}}}_{{{{{{\rm{S}}}}}}}\left({{{{{{\bf{S}}}}}}}_{{{{{{\bf{N}}}}}}}^{{{{{{\bf{t}}}}}}},{{{{{{\bf{A}}}}}}}_{{{{{{\bf{N}}}}}}}^{{{{{{\bf{t}}}}}}}\right)-{\hat{{{{{{\bf{A}}}}}}}}_{{{{{{\bf{N}}}}}}}^{{{{{{\bf{t}}}}}}}{||}}_{1},$$

where ${\hat{{{{{{\bf{A}}}}}}}}_{{{{{{\bf{N}}}}}}}^{{{{{{\bf{t}}}}}}}$ are the ground truth rectified actions of each agent at time $t$, ${|\cdot|}_{1}$ represents the sum of element-wise absolute distance. Since the action rectification also involves complex interactions between agents, we also use the Transformer as the backbone of the safety mapping network. Similar to the behavior modeling network, each agent is considered as an individual token, and the Transformer is trained to predict the residue between the rectified and the reference control. After training, the pretrained safety mapping network will be fixed and embedded into the framework, therefore, the whole pipeline can be trained end-to-end as shown in Fig. 2a.

By incorporating the safety mapping network, we can mitigate the inevitable modeling error of the behavior modeling network in safety-critical situations. We showed that the safety mapper significantly reduces the modeling error (e.g., measured by crash rate) by several orders of magnitude in Ablation studies, while such behavior is extremely difficult for existing data-driven approaches to master due to the “curse of rarity” issue discussed previously. Also, it helps to decouple the safety objective when training the behavior modeling network and let it focus on realistic multi-agent interaction modeling. It should be noted that the proposed method is not limited to the chosen physics-based rule. Different safety guards proposed recently can also be used, for example, safety envelope-based methods^62^, potential force field-based methods^63,64^, online verification methods^65^, etc.

### Generative adversarial training

To further improve the realism of the generated trajectories and tackle the distribution shift issue, generative adversarial training is adopted when training the behavior modeling network. The key to the generative adversarial training is a minimax two-player game under which two networks will contest with each other and force the generated data to be indistinguishable from real ones^42^. During the training, we rollout forward the simulation for several steps and assume the generated trajectories can be easily differentiated from real ones if they exhibit unrealistic patterns (e.g., offroad or other distribution shift behaviors). To this end, we introduce a discriminator network – a multi-layer perceptron network, which takes in the state trajectories of an agent and is trained to distinguish whether the input is sampled from the real-world dataset or from the simulation. Meanwhile, we force the behavior modeling network to capture the true distribution of real trajectories and make generated data indistinguishable from the discriminator side. In this way, the adversarial loss can be backpropagated to the behavior modeling network to further improve the modeling fidelity.

Suppose ${{{{{\rm{D}}}}}}$ represents the discriminator network, $\hat{{{{{{\bf{S}}}}}}} \sim {p}_{R}({{{{{{\bf{S}}}}}}}_{{{{{{\bf{N}}}}}}}^{{{{{{\bf{t}}}}}}})$ represents a trajectory sampled from the real-world data distribution, and ${{{{{\bf{S}}}}}} \sim {p}_{G}({{{{{{\bf{S}}}}}}}_{{{{{{\bf{N}}}}}}}^{{{{{{\bf{t}}}}}}})$ represents a trajectory generated from the simulation. We follow a standard adversarial training pipeline and define the adversarial loss functions as follows:

17 $${{{{{{\rm{L}}}}}}}_{{{{{{\rm{adv}}}}}}}\left({{{{{{\rm{F}}}}}}}_{{{{{{\rm{M}}}}}}},{{{{{\rm{D}}}}}}\right)={E}_{\hat{{{{{{\bf{S}}}}}}} \sim {p}_{R}\left({{{{{{\bf{S}}}}}}}_{{{{{{\bf{N}}}}}}}^{{{{{{\bf{t}}}}}}}\right)}\left[{{\log }}\,{{{{{\rm{D}}}}}}\left(\hat{{{{{{\bf{S}}}}}}}\right)\right]+{E}_{{{{{{\bf{S}}}}}} \sim {p}_{G}\left({{{{{{\bf{S}}}}}}}_{{{{{{\bf{N}}}}}}}^{{{{{{\bf{t}}}}}}}\right)}\left[{{\log }}\left(1-{{{{{\rm{D}}}}}}\left({{{{{\bf{S}}}}}}\right)\right)\right].$$

During the training process, since all components are differentiable, the networks ${{{{{{\rm{F}}}}}}}_{{{{{{\rm{M}}}}}}}$ and ${{{{{\rm{D}}}}}}$ can be alternatively updated under a unified objective. By combining the loss function (Eq. (9)) of the behavior modeling network ${{{{{{\rm{F}}}}}}}_{{{{{{\rm{M}}}}}}}$, our final objective function is defined as follows:

18 $${{{{{{\rm{F}}}}}}}_{{{{{{\rm{M}}}}}}}^{*},{{{{{{\rm{D}}}}}}}^{*}={{\arg }}\mathop{{{\min }}}\limits_{{{{{{{\rm{F}}}}}}}_{{{{{{\rm{M}}}}}}}}\mathop{{{\max }}}\limits_{{{{{{\rm{D}}}}}}}\left\{{{{{{{\rm{L}}}}}}}_{{{{{{\rm{M}}}}}}}\left({{{{{{\rm{F}}}}}}}_{{{{{{\rm{M}}}}}}}\right)+\beta \,{{{{{{\rm{L}}}}}}}_{{{{{{\rm{adv}}}}}}}\left({{{{{{\rm{F}}}}}}}_{{{{{{\rm{M}}}}}}},{{{{{\rm{D}}}}}}\right)\right\}.$$

where ${{{{{{\rm{F}}}}}}}_{{{{{{\rm{M}}}}}}}$ tries to minimize this objective while ${{{{{\rm{D}}}}}}$ tries to maximize it. $\beta$ is a pre-defined hyperparameter for balancing the weights between the two loss terms.

### Network architecture

The network architecture of the behavior modeling network is shown in Fig. 10a. Each road user is considered as a token and the input to the network is its historical trajectory, i.e., position (x, y coordinates) and heading (cosine and sine of heading), of all road users within the historical time window. The number of historical steps at the input is set to $\tau=5$ with a resolution of 0.4 seconds per step. Then, the input will pass through the frequency encoding^61^ layer, in which we apply a set of sine and cosine basis functions that project the vectors to high-dimensional space to improve capturing high-frequency variation in the state spaces. Suppose $\gamma$ defines a mapping function from ${R}^{1}$ to ${R}^{2L+1}$, where $L$ is the order of frequencies, we use $L=4$ in the study. The input embedding layer is a fully connected layer that converts the state dimension to the Transformer hidden layer dimension. Then a set of ($N=4$) standard BERT^41^ transformer layers is stacked together. The dimension of the hidden layer in the Transformer block is 256, the number of heads in multi-headed attention layers is 4, the dimension of intermediate layers in the position-wise feed-forward net is 512, the probability of dropout of various hidden layers is 0, and the probability of dropout of attention layers is 0. Then, the output from the Transformer will pass through prediction heads (single layer MLP with 256 neurons) to generate the final output. In practice, we directly predict the states of each vehicle rather than actions for simplicity. Therefore, the output of the behavior modeling network is the predicted trajectory, i.e., stochastic position (one prediction head for the mean of position, one prediction head for the variance of position, and one prediction head for deterministic heading), of each vehicle in the prediction time horizon. The number of prediction steps at the output is set to $\kappa=5$ with a resolution of 0.4 seconds per step.

The discriminator is a four-layer MLP with dimensions 1024*512*256*1. The activation function is LeakyReLU with a slope equal to 0.2. The input of the discriminator is the trajectory either from the behavior modeling network or the real-world sample and the output is a scalar value. Similar to the behavior modeling network, frequency encoding is applied before passing through the MLP.

The network architecture of the safety mapping network is the same as the behavior modeling network. The input of the network is also the position and heading of all vehicles. The safety mapping network is performed frame-by-frame, so the input includes only the vehicle state at the current step. The output of the safety mapping network is the position and heading after rectifications that project the unsafe state to the nearest safe one.

## Supplementary information

Supplementary Information

Supplementary Movie #1

Supplementary Movie #2

Supplementary Movie #3

Supplementary Movie #4

## Data Availability

The Ann Arbor roundabout dataset that we used to train NeuralNDE is publicly available at https://github.com/michigan-traffic-lab/Learning-Naturalistic-Driving-Environment. The ground-truth crash type, crash severity distributions, and police crash reports are available at https://www.michigantrafficcrashfacts.org/. The background image of the simulated Ann Arbor environment is from Google Maps. The rounD dataset that we used to train NeuralNDE is publicly available at https://www.round-dataset.com/ for non-commercial usage. The background image of the simulated rounD environment is also from the rounD dataset.  Source data for figures are provided with this paper.

## Source data

Source Data

## Code availability

The SUMO simulation platform is publicly available at https://www.eclipse.org/sumo/. The source code used to analyze experiment results and generate figures is publicly available at https://github.com/michigan-traffic-lab/Learning-Naturalistic-Driving-Environment.
