# Supplementary material for: Learning naturalistic driving environment with statistical realism
Source: Nat Commun. 2023 Apr 11;14:2037. doi: 10.1038/s41467-023-37677-5 (PMC10090144; doi:10.1038/s41467-023-37677-5)
Supplement: Supplementary file 1 — Supplementary Information [file 41467_2023_37677_MOESM1_ESM.pdf]

Supplementary Materials

Title

Learning Naturalistic Driving Environment with Statistical Realism

Authors

Xintao Yan[1,6], Zhengxia Zou[1,4,6], Shuo Feng[1,2,5], Haojie Zhu[1], Haowei Sun[1], Henry X. Liu[1,2,3,*]

Affiliations

[1]Department of Civil and Environmental Engineering, University of Michigan, Ann Arbor, MI, USA
[2]University of Michigan Transportation Research Institute, Ann Arbor, MI, USA
[3]Mcity, University of Michigan, Ann Arbor, MI, USA
[4]Present Address: School of Astronautics, Beihang University, Beijing, China
[5]Present Address: Department of Automation, Tsinghua University, Beijing, China
[6]These authors contributed equally: Xintao Yan, Zhengxia Zou
*Corresponding Author, henryliu@umich.edu

1. Supplementary information for experiments

a. Dataset

The Ann Arbor roundabout dataset (abbreviated as AA dataset) is collected at State St. – W Ellsworth Rd. intersection, Arbor, Michigan. A roadside camera-based perception system[1,2] is deployed for real-time traffic object detection, localization, and tracking to collect vehicle trajectory data. For training purposes, we used data collected on May 2nd, 2021, from 10:00 to 17:00, including around 17,000 road users. For each vehicle, the data includes its position and heading information at 2.5 Hz. We excluded frames that involve pedestrians, cyclists, and trailers since there are only a few frames that include these agents and the data size is limited for training. It should be noted that the proposed method can handle diverse road users (e.g., pedestrians) and model their interactions if the data is sufficient. For validation purposes, we used crash data from large-scale trajectories and police crash reports[3] to obtain ground-truth safety-critical events statistics (e.g., crash rate and crash type distribution).

The rounD dataset[4] is collected at several different locations with high accuracy tracking of around 13,000 road users at high frequency (25Hz). We chose the roundabout with most of the data and it is located at Neuweiler, Aachen. Similar to the AA dataset, we also exclude frames that involve pedestrians, cyclists, and trailers for training.

b. Experiment settings

In this study, we assume all vehicles have an identical size with 3.6 meters in length and 1.8 meters in width. Note that the proposed method can be easily extended to handle different vehicle sizes by incorporating length and width in the input data. We simulate approximately 15,000 hours of simulation to record data and generate simulated statistics to validate the statistical realism of the NeuralNDE, where all data are used for calculating crash-related metrics and 100 hours of data are used for other metrics.

c. Evaluation metrics

The Ann Arbor roundabout ground-truth crash rate is obtained based on data from August to mid-November 2021 for around 75 days from 7:00-19:00. There were 14 crashes in this roundabout with a total vehicle travel distance of 1.16×10^5 kilometers. Therefore, the empirical crash rate ground-truth is 1.21×10^{-4} crash/km. The ground-truth crash type and crash severity distributions are queried from the Michigan Traffic Crash Facts[3] dataset whose data is directly from police crash reports. We use data from 2016-2020, and there are a total of 520 crashes at this roundabout with the crash type distribution as shown in Supplementary Fig. 1b. When we calibrate the NeuralNDE, we consider the crash type that is greater than 5%, which includes angle, rear-end, and sideswipe crashes. For the crash severity, we use the worst injury of all involved occupants in the crash as the ground truth. Of the 520 crashes, 498 were non-injury crashes, 22 were minor injuries, and zero were serious and fatal crashes. We have no access to the ground-truth crash rate, crash type distribution, and crash severity data of the rounD roundabout.

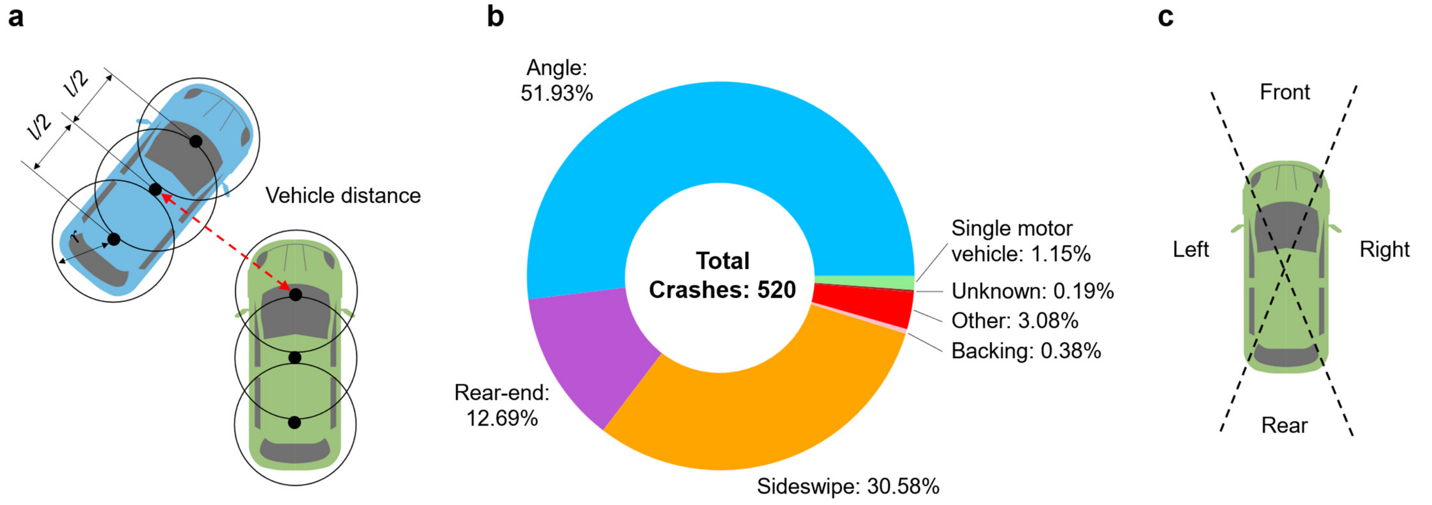

Supplementary Figure 1. a, Vehicle distance illustration. **b,** Ground-truth crash type distribution. Adopted from Michigan Traffic Crash Facts[3]. **c,** Vehicle relative position for crash type classification.

To determine the crash type of a simulated collision, we follow the definition of the National Highway Traffic Safety Administration[5] and consider the state of collision vehicles at the crash moment. Specifically, we consider the relative position and relative heading of the two colliding vehicles. There are four potential relative positions, i.e., front, left, right, and rear, of two colliding vehicles, as shown in Supplementary Fig. 1c. The relative heading of two vehicles is between 0 to 180 degrees, where 0 degrees means two vehicles are heading the same direction and 180 degrees means the opposite direction. We define a crash as rear-end if the relative position is rear or front, and the relative heading is smaller than 40 degrees. A sideswipe crash is when the relative position is left or right, and the relative heading is smaller than 30 degrees or greater than 150 degrees. A head-on crash is when the relative position is front, and the relative heading is greater than 90 degrees. Other crashes are considered angle crashes.

To calculate the change in velocity (Delta-V) based on the conservation of momentum, the collision is assumed to be a perfectly inelastic collision and the vehicles have the same mass. Therefore, the change in velocity can be obtained based on the difference between the impact speed vector and the separation speed vector. For example, consider a rear-end crash, where the front vehicle is initially stationary and the rear-end collision vehicle is traveling at 30 mph. Then, the separation speed of the two vehicles will be 15 mph, and the Delta-V of both vehicles are 15 mph. Many existing studies investigated the relationship between Delta-V and occupant injury level, we follow their found thresholds[6] to measure the crash severity. Specifically, in side impact crashes (e.g.,

angle crash), there is no injury if Delta-V is smaller than 8 mph, minor injury if Delta-V is between 8 and 14 mph, serious injury if Delta-V is between 14 and 24 mph, and fatal injury if Delta-V is greater than 24 mph. For frontal impact crashes (e.g., rear-end crash), the corresponding thresholds are no injury (0,11] mph, minor injury $(11, 23]$, serious injury $(23{,}34]$, and fatal injury $(34, \infty)$.

d. SUMO simulation settings

We compare the proposed method with SUMO[7] - a widely used simulation platform for microscopic traffic behaviors. The map is obtained from the OpenStreetMap[8]. For each episode, the simulation duration and time resolution are the same as NeuralNDE. The Sublane-Model is used to improve the simulation fidelity since by default vehicle lane changes are performed instantly and vehicles are always staying on the centerline of the road in SUMO. The lateral resolution is set as $0.25m$ for the continuous lane-change behavior. The Intelligent Driver Model (IDM)[9] and SL2015 model are used as the car-following model and lane-changing model, respectively. The SUMO default SL2015 lane-changing model is used with default parameters. The IDM model considers both impact of the desired speed and the desired bumper-to-bumper range on the longitudinal behavior, as shown below

$$a(t) = a_{max}\left[1 - \left(\frac{v(t)}{v_0}\right)^{\delta} - \left(\frac{s^{\star}(t)}{s(t)}\right)^{2}\right], \tag{1}$$

where a_{max} is the maximum acceleration, v_0 is the desired speed, $v(t)$ and $s(t)$ are the velocity and the bumper-to-bumper range at the current time step t, δ is an exponent parameter and $s^{\star}$ is the desired bumper-to-bumper range which can be calculated as

$$s^{\star} = s_0 + \max\left(0, v(t) \cdot T - \frac{v(t) \cdot \Delta v(t)}{2\sqrt{a_{max} \cdot a_{comf}}}\right), \tag{2}$$

where s_0 is the minimum range at standstill, T is the desired time headway, $\Delta v(t)$ is the speed difference and a_{comf} is the comfortable deceleration. The model parameters are set based on the data and common practice as follows: $v_0 = 12.5m/s$, $T = 1.0s$, $s_0 = 2m$, $a_{max} = 9.0m/s^2$, $a_{comf} = 4.5m/s^2$, $\delta = 4$. Note that different sets of parameters are examined and results with the best performance are used and reported in the paper.

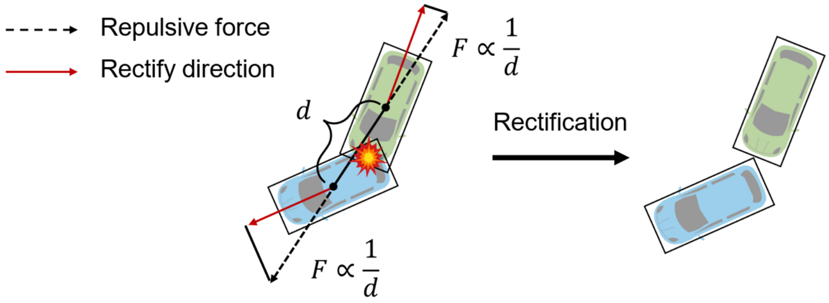

Supplementary Figure 2. Illustration figure of the physics-based safety mapping rule to guide vehicle behavior in safety-critical situations.

e. Training details

We train the safety mapping network by using the RMSprop[10] optimizer. We set the batch size to 64 and the learning rate to 0.0001. The learning rate is reduced to its 0.3 every 600 epochs. The training took around 20 days on an Intel i7-10700F CPU and NVIDIA 3070 GPU desktop with a total number of 3,000 training epochs. To cover all potential safety critical patterns, we randomly sampled the vehicle states as input and their ground truth

is generated with a rule-based model. When two vehicles are going to collide, we push them apart by setting a repulsive force between them. The force is projected to the heading direction of each vehicle and rectifies their states until they are not colliding with each other, as illustrated in Supplementary Fig. 2. Each vehicle is considered as 3.8 meters in length and 2.0 meters in width when training the safety mapper, which includes a 0.2 meters buffer compared to the real size. Note that we do not modify the heading of each vehicle and only rectify the position, which is similar to guiding the vehicle to decelerate or accelerate in safety-critical situations to avoid a crash. Instead of directly predicting the rectified states, we train the mapper to generate the residual between the ground truth and the input. The rectification is performed frame-by-frame. The mean absolute error between the predicted position residue and the ground-truth residue is used as the loss function. Since safety-critical situations rarely happen, the residual may follow a sparse pattern where most of the values are close to zero. Therefore, when generating the training data, we balance the ratio between the activated and non-activated output by using heuristic sampling where in each frame, the first 80% of vehicle states are uniformly sampled and the rest 20% are sampled from the neighbor of existing vehicles. We generate 240,000 random frames for each training epoch. During the training phase, the number of tokens (vehicles) is set to a fixed number of 32 considering the batch-wise training efficiency.

When training the behavior modeling network, we freeze the safety mapping network. Both the behavior modeling network and the discriminator are updated jointly by using the RMSprop[10] optimizer. The batch size is set to 32 and the learning rate is set to 0.0001 with decay to its 0.3 every 300 epochs. We set the training token size to 32. When there are fewer than 32 vehicles in the road network, fake vehicle states will be used to pad the input matrix. Data augmentation is applied with Gaussian noise of zero mean and 0.0025 variance for position and 0.000001 for cosine and sine of heading. The number of training epochs is set to 1,500 and the training takes around 3 days on an NVIDIA 3070 GPU desktop. The number of historical steps at the input is set to 5 with a resolution of 0.4s per step. The number of output steps within a single forward pass is set to 5 with the same resolution. In practice, we train the network to predict the states rather than the actions. The state variables include the position (x and y coordinates) and heading (cosine and sine heading) of each vehicle. The loss function is composed of three parts: imitation loss of position, imitation loss of heading, and adversarial loss. The weight of each component is set as 1, 20, and 0.1. The imitation loss of position and heading is calculated by the mean absolute error between the predicted states (predicted x and y coordinates and heading) and ground-truth states at the next 5 steps. The adversarial loss is calculated using the BCEWithLogitsLoss following the general setting of generative adversarial training.

f. Model scalability experiment settings

We use SUMO[7] to generate vehicle trajectory data and use it as the ground truth of NDE for training and validation. We collect around five hours of data to train NeuralNDE models for the intersection and roundabout scenarios. The training settings are the same as previous experiments using AA and rounD datasets. During the inference time, the intersection and roundabout areas are controlled by NeuralNDE, and the transition area in between is controlled by model-based methods. Since there is no crash in NDE (i.e., SUMO), the acceptance probability of the crash critic module is set to zero. We also apply the safety mapping rule that used to train the safety mapping network to the whole simulation environment including the transition area to further guarantee the safety of the simulation. We simulate the whole network for around 100 hours and collect the data to validate the performance. The simulation resolutions and metrics definitions are the same as in previous experiments. The PET is collected for vehicles within the roundabout circle and intersection. The instantaneous speed for the intersection scenario is collected for all vehicles in the area.

Supplementary Table 1. Ablation study results

	Backbone network	Safety Mapping Network	Adversarial Training	Conflict Critic Module	Instantaneous Speed ↓	Distance ↓	Yielding Distance ↓	Yielding Speed ↓	Crash Rate (crash/km)	Crash Type ↓
1	MLP	✓	✓	✓	0.119	0.076	0.194	0.073	7.81×10^{-4}	0.130
2	LSTM	✓	✓	✓	0.092	0.055	0.090	0.092	1.76×10^{-4}	0.113
3	Transformer	✗	✓	N/A	**0.037**	**0.030**	0.036	0.033	2.34×10^{-1}	0.333
4	Transformer	✓	✗	✓	0.038	0.032	**0.033**	0.040	1.29×10^{-4}	0.041
5	Transformer	✓	✓	✗	0.040	0.032	0.036	**0.032**	1.22×10^{-5}	0.580
6	Transformer	✓	✓	✓	0.040	0.031	0.037	0.033	$\mathbf{1.25 \times 10^{-4}}$	**0.019**

N/A in the table means not applicable.

2. Supplementary results

a. Ablation study

To examine the effectiveness of each module of the proposed framework, we conduct ablation studies in this section. Each model setup was run for approximately 10,000 hours of simulation. The results are shown in Supplementary Table 1. Besides the crash rate column, other values in the table denote the Hellinger distance between the simulated distribution and ground-truth distribution. The smaller the Hellinger distance for the metrics, the better the model performance. For the crash rate, the closer to the real-world ground truth (1.21×10^{-4} crash/km), the better the performance.

To investigate the effects of the behavior network backbone, a two-layer Multilayer Perceptron (MLP) (hidden dimension equals 256) with batch normalization layers and Relu activation function is compared. The main difference between the MLP and Transformer is that the Transformer utilizes the self-attention mechanism in its architectural design. By formulating the road agents as individual tokens, the self-attention mechanism in Transformer is naturally capable of characterizing inter-token interaction between agents. The result shows that the performance of the Transformer backbone is significantly better in all metrics compared to the MLP backbone. We also compared the Long Short-Term Memory (LSTM) network (two layers, hidden dimension equal to 256) as the backbone architecture. To model the interactions between agents, the LSTM module is embedded in a Seq-to-Seq framework[11] as a recurrent unit. This design allows the network to handle the interactions among all input agents instead of only historical ones. The results show that Transformer can achieve better performance in modeling vehicle interactions.

We also examine the importance of the safety mapping network. The conflict critic module is not applicable without the safety mapping network. From the results, we can find the crash rate is extremely unrealistic and multiple magnitudes higher than the ground truth. This result validates the performance of the proposed safety mapping network that significantly reduces the modeling error in safety-critical situations. The model exhibits good performance in other metrics since it does not consider safety performance and only optimizes to imitate normal driving behaviors.

Finally, we demonstrated the significance of the conflict critic module and the adversarial training. We cannot control the generation process of safety-critical events without the conflict critic module, therefore, we cannot obtain accurate crash rate and crash type distribution. Without adversarial training, we found that the crash rate and crash type distribution will be degraded. Table 1 demonstrates that the proposed model exhibits the overall best performance considering all evaluation metrics.

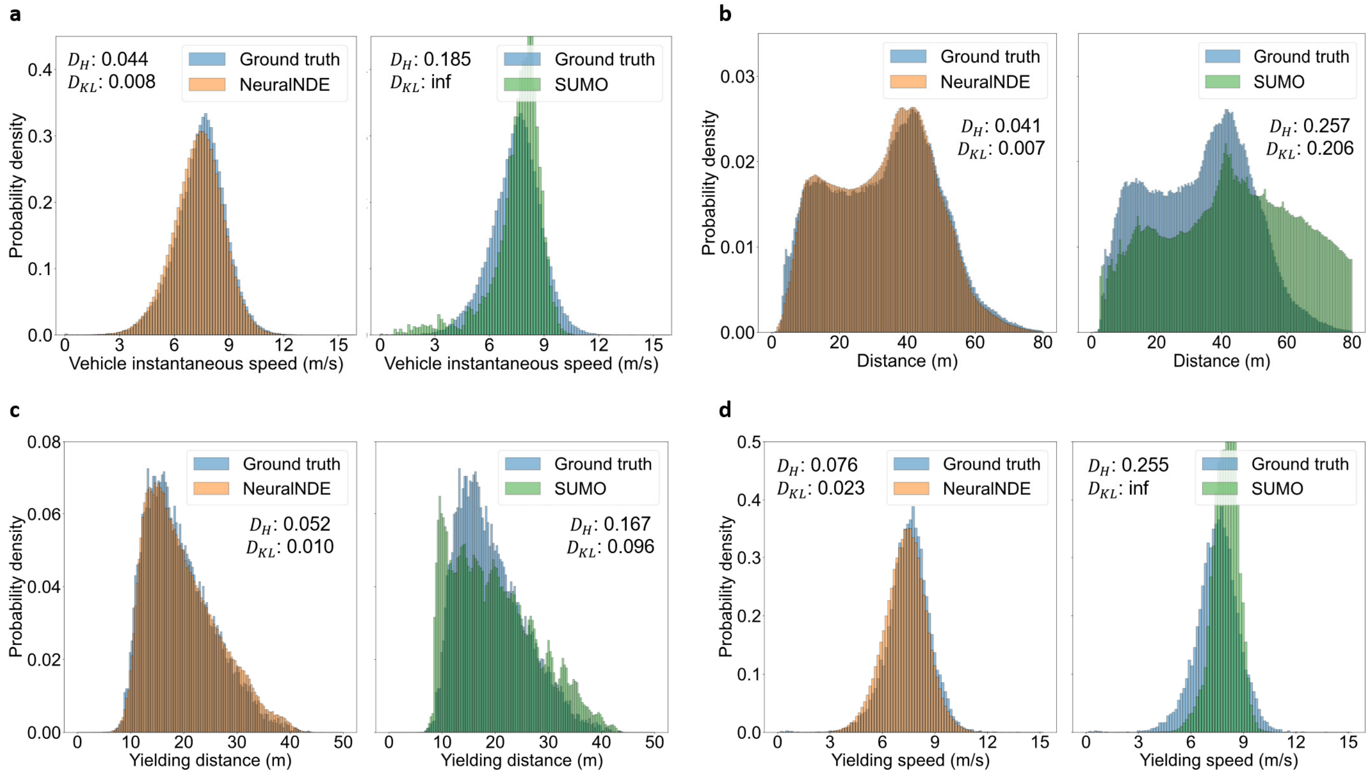

Supplementary Figure 3. Statistical realism of NeuralNDE using rounD dataset. a, Vehicle instantaneous speed distribution. **b,** Vehicle distance distribution. **c,** Yielding distance distribution: distance between the yielding vehicle and its nearest conflicting vehicle. **d,** Yielding speed distribution: speed of the nearest conflicting vehicle. D_H and D_{KL} denote the Hellinger distance and the KL-divergence, respectively.

b. NeuralNDE results of rounD dataset

The NeuralNDE results using the rounD dataset are shown in Supplementary Fig. 3. From the results, we can find that NeuralNDE can achieve statistical realism and significantly outperform existing methods. We only show normal driving behavior statistics since the safety-critical driving behavior ground truth, e.g., crash and near-miss data, is unavailable.

Supplementary references

1. Zou, Z., Zhang, R., Shen, S., Pandey, G., Chakravarty, P., Parchami, A., & Liu, H. X. Real-time Full-stack Traffic Scene Perception for Autonomous Driving with Roadside Cameras. *IEEE International Conference on Robotics and Automation (ICRA)* (IEEE, 2022).
2. Zhang, R., Zou, Z., Shen, S., & Liu, H. X. Design, implementation, and evaluation of a roadside cooperative perception system. *Transportation Research Record* (2022).
3. Michigan Traffic Crash Facts. https://www.michigantrafficcrashfacts.org/.
4. Krajewski, R., Moers, T., Bock, J., Vater, L., & Eckstein, L. The rounD dataset: a drone dataset of road user trajectories at roundabouts in Germany. *IEEE International Conference on Intelligent Transportation Systems (ITSC)* 1-6 (IEEE, 2020).
5. National Highway Traffic Safety Administration. 2020 FARS/CRSS coding and validation manual. Report No. DOT HS 813 251 (2022).
6. Richards, D. C. "Relationship between speed and risk of fatal injury: pedestrians and car occupants." (2010).
7. Lopez, P. et al. Microscopic traffic simulation using SUMO. *International Conference on Intelligent Transportation Systems (ITSC)* 2575-2582 (IEEE, 2018).

8. OpenStreetMap contributors. https://planet.osm.org (2017).
9. Treiber, M., Hennecke, A., & Helbing, D. Congested traffic states in empirical observations and microscopic simulations. *Phys. Rev. E*. **62**, 1805 (2000).
10. Hinton, G., Srivastava, N., & Swersky, K. Neural networks for machine learning lecture 6a overview of mini-batch gradient descent. *Cited on*, **14**, 2 (2012).
11. Sutskever, I., Vinyals, O., & Le, Q. V. Sequence to sequence learning with neural networks. Advances in neural information processing systems, 27 (2014).
